# Effect of weak measurement on entanglement distribution over noisy channels

**DOI:** 10.1038/srep22408

**Published:** 2016-03-03

**Authors:** Xin-Wen Wang, Sixia Yu, Deng-Yu Zhang, C. H. Oh

**Affiliations:** 1 College of Physics and Electronic Engineering, Hengyang Normal University, Hengyang 421002, China; 2 Center for Quantum Technologies, National University of Singapore, 2 Science Drive 3, Singapore 117542.

## Abstract

Being able to implement effective entanglement distribution in noisy environments is a key step towards practical quantum communication, and long-term efforts have been made on the development of it. Recently, it has been found that the null-result weak measurement (NRWM) can be used to enhance probabilistically the entanglement of a single copy of amplitude-damped entangled state. This paper investigates remote distributions of bipartite and multipartite entangled states in the amplitudedamping environment by combining NRWMs and entanglement distillation protocols (EDPs). We show that the NRWM has no positive effect on the distribution of bipartite maximally entangled states and multipartite Greenberger-Horne-Zeilinger states, although it is able to increase the amount of entanglement of each source state (noisy entangled state) of EDPs with a certain probability. However, we find that the NRWM would contribute to remote distributions of multipartite W states. We demonstrate that the NRWM can not only reduce the fidelity thresholds for distillability of decohered W states, but also raise the distillation efficiencies of W states. Our results suggest a new idea for quantifying the ability of a local filtering operation in protecting entanglement from decoherence.

It is well known that establishment of quantum entanglement among distant parties is a prerequisite for many quantum information protocols. Moreover, a necessary condition for perfectly implementing these tasks is that the shared entangled states among the users are maximally entangled pure states. In practice, however, unavoidable interactions of the entangled systems with environments during their distributions or storages would result in degradation of the entanglement among the users. In other words, the entanglement resources actually available are usually entangled mixed states, which would decrease the fidelities and efficiencies of quantum information processes.

To accomplish the aforementioned quantum information processing tasks, the communicators need to transform the noisy entangled states into maximally entangled pure states in advance. This raises a problem which is also of theoretical interest: How can maximally entangled pure states be extracted from shared entangled mixed states by local operations? One solution, at least in principle, is to use entanglement distillation protocols (EDPs) which function as distilling a small number of entangled pure or nearly pure states from a large number of entangled mixed states^1^,^2^,^3^,^4^,^5^. This means perfect or nearly perfect entanglement-based quantum information processing would be possible even in noisy environments by utilizing the idea of entanglement purification.

However, the EDPs do not work for the inseparable states whose fidelities or singlet fractions (which quantify how close the states are to maximally entangled states^1^,^6^) are less than some thresholds (e.g., 1/2 for two-qubit states^1^,^2^), except that they have some special forms or are hyperentangled^6^,^7^,^8^,^9^,^10^,^11^. Fortunately, Gisin^12^ discovered that the amount of entanglement of an entangled mixed state could be raised probabilistically by local filtering operations, which has been proven in the experiment^13^. Moreover, local filtering could be used to make trace-preserving local operations assisted by classical communication so as to increase limitedly the fidelities of some low-fidelity entangled mixed states with entanglement unchanged^14^,^15^,^16^,^17^,^18^. These findings enable the entanglement of little-entangled particles (even with fidelities less than the thresholds) to be distillable, because they can be put through local filters, such that their fidelities are over the related thresholds, prior to being subjected to EDPs^19^.

Recently, purification of a single-copy entangled mixed state by local filtering operations has attracted considerable interest^20^,^21^,^22^,^23^,^24^,^25^,^26^,^27^,^28^,^29^,^30^,^31^,^32^,^33^,^34^,^35^,^36^,^37^,^38^,^39^, due to the fact that it does not involve multiparticle collective operations on multiple copies of source states and thus may reduce the experimental difficulty, as well as can act as a complement to entanglement distillation. The null-result weak measurement (NRWM, a local filtering operation)^40^ is widely used to enhance the entanglement of various decohered states in amplitude-damping (AD) or generalized AD environments^20^,^21^,^22^,^23^,^24^,^25^,^26^,^27^,^28^. The experimental viability of implementing a NRWM and its reversal^21^,^41^,^42^,^43^,^44^,^45^,^46^,^47^ indeed makes it an elegant approach to protecting entanglement. However, the filtering method cannot be applied for the direct production of entangled pure states^48^,^49^. To obtain maximally entangled pure states for perfect remote quantum information processing, EDPs are required. Then, a question arises, namely, is the NRWM beneficial to entanglement distribution among distant parties in terms of the efficiency of extracting maximally entangled states, although it can improve with a certain probability the entanglement of each source state (initial noisy entangled state) of the EDP? This paper is addressing such an issue.

We consider entanglement distribution over AD channels. The aim of the users is to share maximally entangled states. As mentioned before, to achieve remote distribution of maximally entangled states, we resort to the entanglement distillation. In previous literatures^20^,^21^,^22^,^23^, the NRWM was introduced to raise the amount of entanglement of a single-copy decohered state in AD environments. We here investigate the impact of the NRWM on entanglement distribution efficiencies (i.e., the efficiencies of distilling maximally entangled states) by using it to enhance the entanglement of each decohered state before starting the distillation procedures. We show that NRWMs would decrease distillation efficiencies of bipartite maximally entangled states and multipartite Greenberger-Horne-Zeilinger (GHZ) states^50^. The efficiency (also known as yield in literature) of an EDP is conventionally defined as the ratio of the number of obtained maximally entangled states to that of source states (inputs). Multipartite W-state^51^ distribution, however, exhibits different behaviors and features. That is to say, the NRWM would contribute to increasing the efficiency of W-state distribution with the existing EDP or its generalization and reducing the fidelity threshold for distillability of the decohered W state. Our results indicate that the NRWM is not necessarily helpful to practical entanglement distributions, although it is able to increase the amount of entanglement of a single-copy noisy entangled state, and thus suggest a new approach to quantify the ability of a local filtering operation in protecting entanglement from decoherence.

The rest of this paper is organized as follows. In the Results section, we first demonstrate the uselessness of NRWMs to distributions of bipartite entangled states and multipartite GHZ states, and then discuss the effect of the NRWM on W-state distribution. We offer our conclusions in the Discussion section. Some technical bits are deferred to the Methods section.

## Results

### Bipartite entanglement distribution

The quantum channel considered in this paper is the AD channel. AD decoherence is applicable to many practical qubit systems, including vacuum-single-photon qubit with photon loss, photon-polarization qubit traveling through a polarizing optical fiber or a set of glass plates oriented at the Brewster angle, atomic qubit with spontaneous decay, and superconducting qubit with zero-temperature energy relaxation. The action of the AD channel on a qubit *l* can be described by two Krauss operators^52^


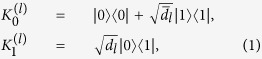


where *d*_*l*_ stands for the damping rate satisfying 0 ≤ *d*_*l*_ < 1 and 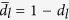. The AD channel is trace preserving, that is, 
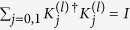
. Note that *d* = 0 denotes the noise-free case, and it will not be considered in the following context.

Assume the initial entangled state to be distributed to Alice and Bob is a 2-qubit Bell state given by


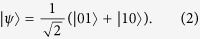


During the process of distributing or storing, the two qubits would experience AD decoherence with decoherence strength 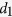 and 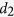, respectively. The original entangled pure state then degrades into a mixed state


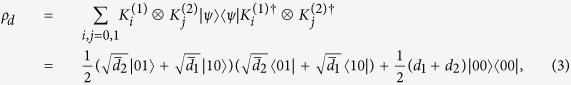


where the superscripts of *K*_*i,j*_ denote the qubit indices. The concurrence (a universal entanglement measure for 2-qubit states^53^) of *ρ*_*d*_ is


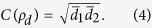


As claimed and demonstrated in recently reports^20^,^21^,^22^,^23^, the concurrence of the decohered state *ρ*_*d*_ can be improved probabilistically by performing locally each qubit a weak measurement, accompanied by a bit flip operation before and after the weak measurement, respectively. The weak measurement is a kind of measurement that does not totally collapse the measured system. Practically, the weak measurement on a qubit can be done by monitoring its environment using a detector^21^,^41^,^42^,^43^,^44^,^45^,^46^,^47^. Whenever the detector registers an “excitation”, one knows that the qubit has totally collapsed into its ground state; if, however, there is no “excitation” (null result), one knows that the qubit state is just renormalized. Mathematically, such a measurement can be described by two positive operators


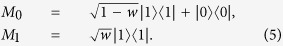


If we discard the outcome of 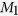, then 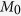 denotes the NRWM (null-result weak measurement) of strength *w* (0 ≤ *w* < 1), that partially collapses the system to the ground state. The NRWM in fact uses post-selection to selectively map the state of a qubit. If no outcome is discarded, the two operators 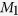 and 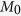 will describe a noisy effect. Considering that a flip operation 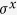 (conventional Pauli operator) is preformed on the system before and after the NRWM 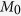, respectively, the total process can be described by the operator


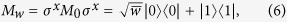


where 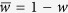. For convenience, 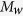 will be directly referred to as the NRWM operator. After Alice (holds the first qubit) and Bob (holds the second qubit) performing NRWMs of strength 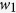 and 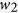 on the entangled pairs, respectively, the state 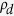 becomes


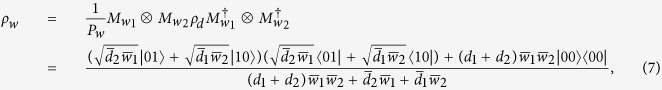


where 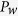 is the probability of getting the outcome of 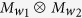, i.e. the probability of successful event, given by


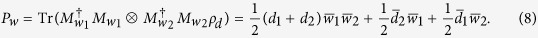


Evidently, 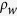 is equivalent to 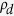 for 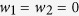 that means no weak measurement is made. Naturally, 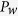 is then equal to 1. The concurrence of 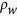 can be calculated as


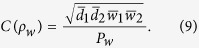


*C*(*ρ*_*w*_) is larger than 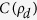 provided that 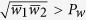. Such a condition can be satisfied for any *d*_1_ and *d*_2_ by choosing suitable 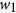 and 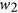. For instance, the inequality always holds for 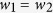. It is easy to see that when *C*(*ρ*_*w*_) → 1 (corresponding to 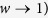, the success probability 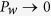.

Although the entanglement established between Alice and Bob was improved by NRWMs, the shared entangled state is still not a maximally entangled pure state that is a prerequisite for some perfect quantum communications (e.g., teleportation). As mentioned before, the filtering operations cannot be, even in principle, applied for the direct production of maximally entangled states^48^,^49^. To obtain maximally entangled states, Alice and Bob need further to utilize EDPs.

Next, we investigate whether the NRWM can help Alice and Bob to raise the efficiency of getting maximally entangled states by transforming the decohered state *ρ*_*d*_ to *ρ*_*w*_ using NRWMs before starting the EDP. We will employ two EDPs, both of which enable bipartite maximally entangled pure states to be extracted from finite copies of *ρ*_*w*_ or *ρ*_*d*_ (corresponding to 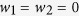 in 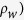. The first EDP will be called a two-copy EDP, because each round of distillation only involves two copies of input states^6^. The second EDP will be referred to as a bisection EDP, because each round of distillation except the first round divides the pairs of qubits into two blocks of equal length^54^. The bisection EDP is up to now the most efficient theoretical scheme for the amplitude-damped state 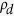 or 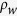
54, although it is much more difficult than the two-copy EDP in the experiment.

### Two-copy EDP

Suppose there is a collection of groups of source entangled pairs 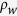. Each group contains two pairs, one as the control pair and the other as the target pair. Each party of Alice and Bob holds one qubit of each pair. The EDP works as follows: (i) Alice and Bob apply, respectively, a local controlled-not (CNOT) gate between the two pairs of each group (i.e., the bilateral CNOT operation^6^), where the control pair comprises the two control qubits and the target one the two target qubits; (ii) they measure locally the target pair in the computational basis 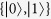; (iii) they keep the control pair if they get the outcomes “11” (this means the success of extracting a maximally entangled state) and “00” (in this case, the control pair can be used for the second round of distillation), and discard it otherwise.

It can be easily verified that if the outcome of this measurement on a given target pair is “11”, then the corresponding control pair is left in the Bell state 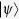 which can be used for faithful teleportation, etc. The probability of this event is


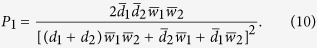


Since each target pair has to be sacrificed for the measurement, the yield from this procedure is 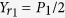. As for the measurement outcome “00” of the target pair, the corresponding control pair is left in the state


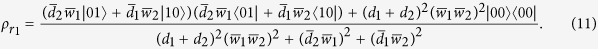


The probability of this event is


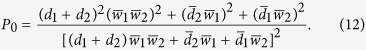


Evidently, two copies of 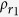 can be used for the second round of distillation following the procedure above. Then after *m* rounds of distillation procedure, the efficiency (total yield) of this EDP becomes


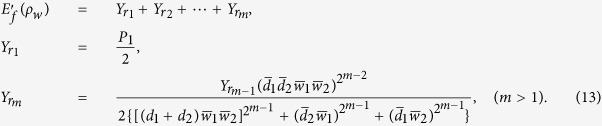


Naturally, for a given EDP, the more entangled the source states are, the higher efficiency would be obtained. As a consequence, the value of 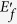 in a general case (i.e., 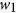 and 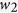 are not simultaneously equal to zero) can always be larger than that of it in the case 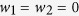 for given 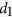 and 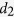, because the source state 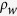 can be more entangled than 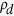.

What will happen when considering the fact that the probability of getting 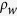 from 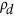 by NRWMs is not one but 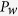 given in Eq. (8)? Under this situation, the efficiency of the above entanglement distribution scheme, with NRWMs being performed in advance on each copy of 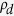, should be


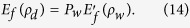


**Figure 1:**
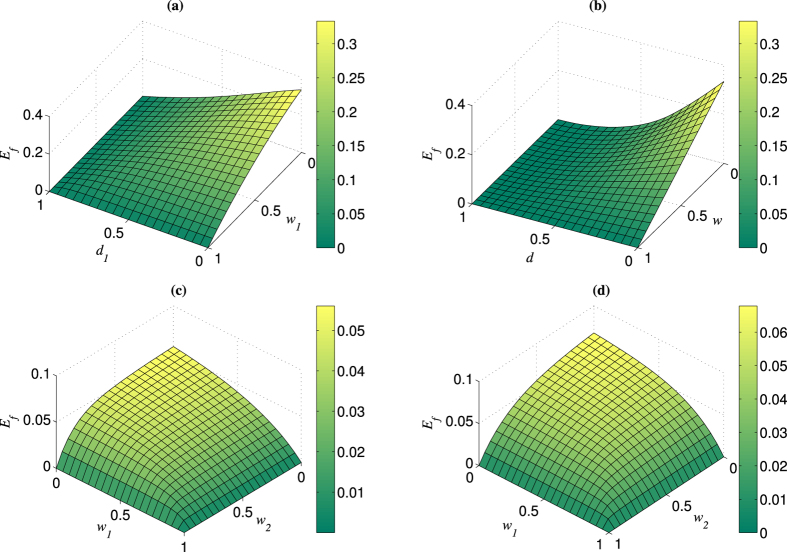
Variation in the bipartite entanglement distribution efficiency *E*_*f*_ with weak measurement strengths (*w*_1_, *w*_2_) and channel damping rates (*d*_1_, *d*_2_). Here the number of rounds is taken 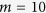. (**a**) *d*_2_ = 0 = *w*_2_; (**b**) *d*_1_ = *d*_2_ = *d* and *w*_1_ = *w*_2_ =*w*; (**c**) *d*_1_ = 0.3 and *d*_2_ = 0.7; (**d**) *d*_1_ = *d*_2_ = 0.5.

That is, the final efficiency is the product of the efficiencies of two stages: filtering and distillation protocol. If one does not recycle the state 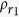 corresponding to the aforementioned measurement outcome “00”, the efficiency 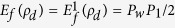. It is easy to prove that 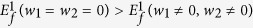 for arbitrarily given 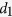 and 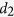. This means that the efficiency of the distillation scheme with NRWM is lower than that of the scheme without NRWM. Such a conclusion is still tenable in the case of any *m* rounds of distillation. As an example, we plot the efficiency 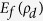 for 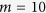 in Fig. 1. It can be seen from Fig. 1 that 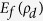 takes the maximum only when 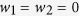 (that means no weak measurement) for any 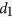 and 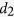. All the above results imply that the NRWM does not increase but decreases the efficiency of bipartite entanglement distribution, i.e., the efficiency of extracting the Bell state 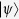 from the amplitude-damped state 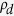. Thus, the NRWM would generate a negative impact on the bipartite entanglement distribution. The same conclusion will be obtained for the bisection EDP as shown in the next subsetion.

**Figure 2:**
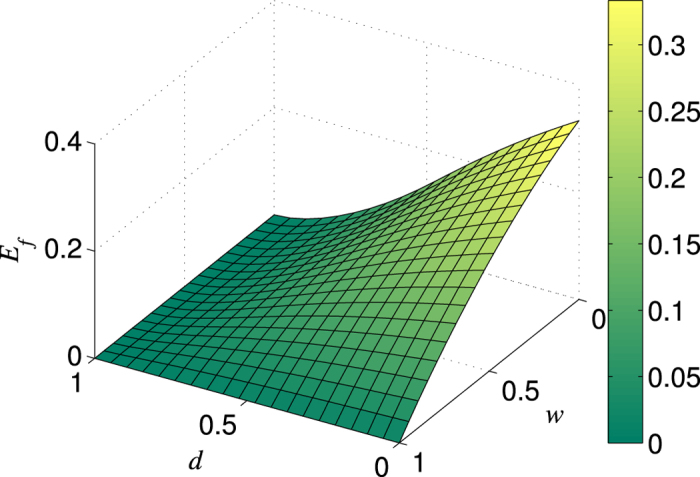
Dependence of the bipartite entanglement distribution efficiency *E*_*f*_ on the weak measurement strength *w* and channel damping rate *d* when using 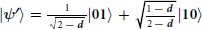 as the initial state and just one qubit of it suffers from the AD noise.

We notice that some 2-qubit partially entangled pure states are more robust than the Bell state 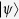 in terms of the singlet fractions of the decohered states when just one qubit interacts with the AD channel^17^,^18^. In this case, the efficiency of establishing maximally entangled states between Alice and Bob may be slightly improved by substituting the input Bell state 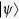 for an appropriate 2-qubit partially entangled pure state. However, it will make no difference to the conclusion that the NRWM would reduce the efficiency of preparing nonlocal Bell states. As an example, we replace the initial Bell state 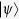 by the nonmaximally entangled pure state 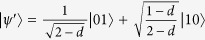 from which the maximum singlet fraction is obtained when only one qubit suffers from the AD noise^17^,^18^. By the same procedure as before and setting 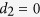 (or 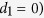, we obtain the final efficiency of establishing Bell states between Alice and Bob, as displayed in Fig. 2. Figure 2 indicates that the no-NRWM-scheme 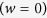 still outperforms the NRWM-scheme (*w* > 0) even using 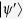 as the initial state.

### Bisection EDP

Let Alice and Bob share *n* copies of state 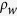, where *n* is the power of two. For simplicity, we assume 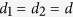 and 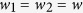. Then 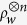 can be conveniently written as


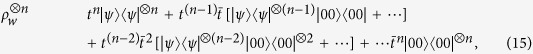


where


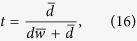



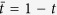
, and “
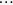
” in each square bracket denotes all permutations of the first term in the square bracket.

Now both Alice and Bob project her/his part of the state 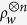 on a subspace spanned by vectors with definite number of “1”. That is, they perform their particles von Neumann measurements given by the sets of projectors


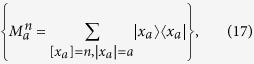



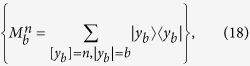


respectively, where 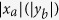 denotes the Hamming weight of the string 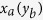 of 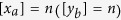 qubits and 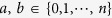. If Alice obtains the measurement outcome 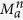 and Bob obtains 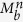, the state of the *n* pairs collapses into


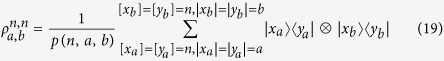


with 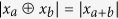 and 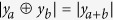, where “
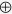
” standing for modulo-2 sum of the bitwise of two strings 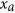 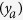 and 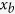 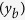, e.g., 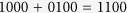. The sign “
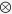
” in the above equation denotes the partition “Alice:Bob” of 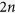 qubits and 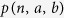 is given by


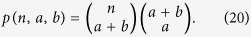


The probability of this event is


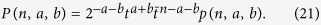


If 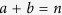, then Alice and Bob share a maximally entangled pure state of the rank


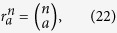


which is equivalent to \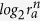 maximally entangled pairs of qubits. If any one of the equalities 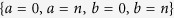 holds, Alice and Bob share a separable state. In the remaining cases, the state 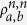 is inseparable in terms of the partition “Alice:Bob”, that is reusable in the second round of distillation. Using the bisection method (Alice and Bob divide the pairs of qubits into two blocks of equal length) in the following rounds of distillation, the total yield of such an EDP starting from the state *ρ*_*w*_ is given by^54^


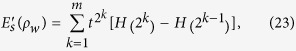


where 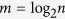 and


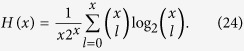


Considering the fact that the probability of obtaining 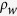 from the original decohered state 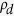 is 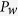 as Eq. (8) with *d*_1_ = *d*_2_ = *d* and 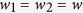, the final efficiency of Alice and Bob sharing maximally entangled pure states should be


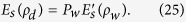


**Figure 3:**
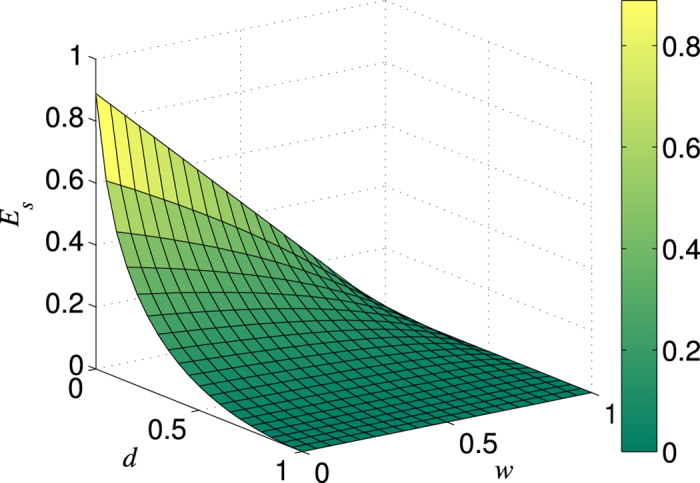
Variation in the bipartite entanglement distribution efficiency *E*_*s*_ with *d* and *w*. Here we take the number of source pairs *n*  = 32.

The efficiency 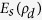 as a function of *d* and *w* with 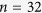 is exhibited in Fig. 3. It can be seen that 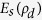 takes the maximum only when 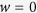 (that means no weak measurement) for an arbitrarily given *d*, and that the larger *w*, the lower 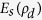. This result further justifies the fact that NRWMs would decrease the efficiency of distributing maximally entangled pairs to two distant parties. Note that although the total yield of the bisection EDP could be further improved by combining one-way hashing method after the first round of distillation^54^, it will not change the conclusion above, due to the fact that the yields of all rounds except the first round of distillation procedure are not related with the weak measurement parameter *w*. In addition, we can see from Figs. 1 and 3 that the decrease of 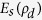 caused by NRWMs in the bisection protocol is larger than that in the two-copy protocol. It implies that the more efficient the EDP is, the larger adverse impact the NRWM will have.

The negative influence of the NRWM on the above-mentioned bipartite entanglement distribution could be partly understood from that as follows. If putting the source states (original noisy states) through local filters prior to starting distillation procedure, then the final entanglement distribution efficiency is the product of the efficiencies of two stages: filtering and distilling. Although the NRWM could increase the yield of the second stage, it will decrease the success probability of the first stage (the probability is one when no weak measurement is performed). The competition of two opposite effects in two stages leads to the result above.

What is the case for multipartite entanglement distribution? In the next two sections, we will elucidate such a problem by discussing the impact of the NRWM on GHZ-state and W-state distributions, respectively.

### Multipartite GHZ-state distribution

In this section, we investigate the effect of the NRWM on the efficiency of GHZ-state distribution in the AD environment based on multipartite EDPs. The existing GHZ-state distillation protocols only deal with “Werner-type” or GHZ-diagonal states and work in asymptotic ways^55^,^56^,^57^,^58^. It is not clear whether these protocols can be applied to amplitude-damped GHZ states which are not GHZ-diagonal states. We here present an efficient GHZ-state distillation protocol which is suitable for the scenario considered here. This protocol works out of asymptotic way, and can be regarded as a generalization of the aforementioned two-copy EDP for two qubits to a multipartite case. For clarity and simplicity, we here just discuss the case of 3-qubit GHZ-state distillation and distribution, and the obtained results can be extended to *N*-qubit GHZ states.

Suppose the initial 3-qubit GHZ state to be distributed to three distant parties is in the form


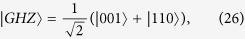


where the three qubits are not all parallel. After each qubit independently suffering the AD decoherence during the process of distribution or storage, the state 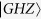 will degrade into an entangled mixed state denoted by *ρ*′_*d*_. If assume the decoherence strength of every qubit is the same, the noisy GHZ state is in the form


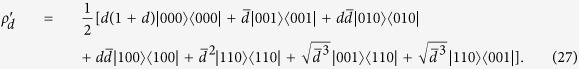


We now perform each qubit a NRWM described by the operator 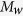 given in (6). Under the successful event, the noisy GHZ state becomes 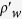 which can be obtained by multiplying each ‘
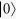
’ or ‘
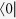
’ of 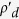 by the factor 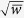 (e.g.,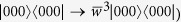. The success probability is


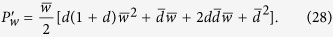


According to the analysis in ref. 25, 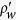 could be more entangled than 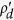 in terms of the measures of negativity and multipartite concurrence, and thus the fidelity of the former could also be higher than that of the latter^29^.

**Figure 4:**
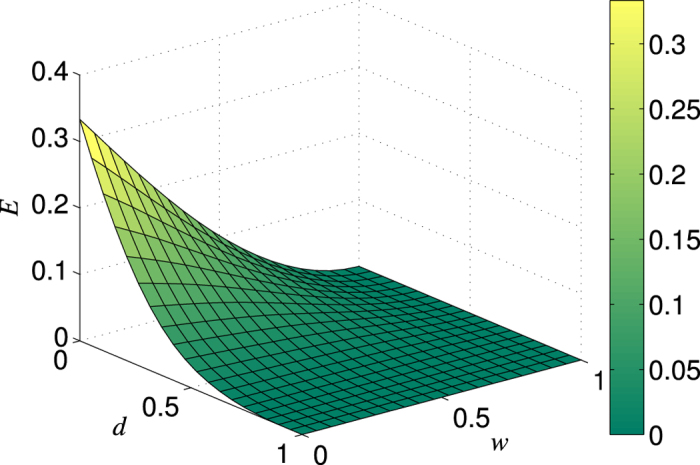
Dependence of the 3-qubit GHZ-state distribution efficiency *E* on the weak measurement strength *w* and channel damping rate *d*.

However, we shall show that the NRWM is not good for distilling pure GHZ states from noisy GHZ states. The proposed distillation protocol works as follows: (i) All the three parties take two copies of the input state 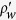 (or 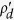 with 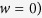; (ii) each one labels the first qubit control and the second target and perform a CNOT-gate operation on his/her two qubits; (iii) they measure their target qubits in the basis 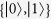; (iv) they keep the control qubits if they get the outcome “111” (this means the success of extracting the pure GHZ state 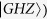 or “000” (in this case, the control copy can be used for the second round of distillation), and discard it otherwise. Following the same processing as the bipartite two-copy EDP introduced above, we obtain the formula of the final distribution efficiency of the GHZ state 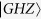,


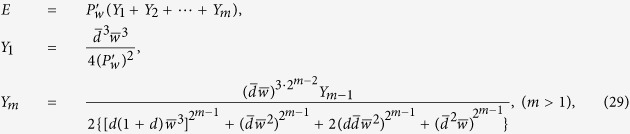


where *m* denotes the number of rounds. The specific dependence of the efficiency *E* on the parameters *d* and *w* for 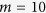 is exhibited in Fig. 4. From Fig. 4, we can see that *E* takes the maximum value only when 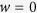 (that means no weak measurement) for any *d*. This result means that the NRWM is bad for the distribution of the 3-qubit GHZ state. We believe the conclusion is also applicable to *N*-qubit GHZ states. The origin of the negative influence of the NRWM on the GHZ-state distribution may be the same as that of Bell-state distribution.

### Multipartite W-state distribution

Next, we discuss the role of the NRWM in the distribution of W states and show different phenomena from that observed before. The W state is a peculiar type of multipartite entangled state, and has attracted particular interest on its properties and applications^51^,^59^,^60^,^61^,^62^,^63^,^64^. Entanglement distillation of 3-qubit dephased and depolarized W states was studied in ref. 65. A EDP for the 3-qubit amplitude-damped W state has been proposed in ref. 66. Here, we show that the EDP in ref. 66 can be generalized to *N*-qubit W states, and that the yields of W-state distillation schemes could be improved by the aforementioned NRWM. Moreover, the fidelity thresholds for distillability of decohered W states could be reduced to near zero.

Suppose the perfect *N*-qubit W state


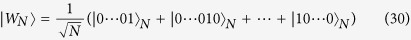


is distributed to *N* parties (Alice, Bob, Charlie, 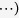, but suffers typical decoherence as described by the local AD channel with the same damping rate *d*. Then the *N* parties initially share a noisy W state given by


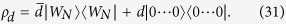


The fidelity of this noisy W state relative to the original pure W state is 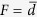.

We show that the fidelity of 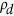 can be improved probabilistically by performing each qubit a NRWM described in Eq. (6). After each of the *N* parties performing a NRWM on the qubit he/she holds with a successful event, the state 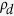 becomes


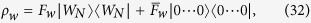


where the fidelity 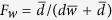 is the same as *t* in Eq. (16) and 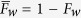. The success probability is


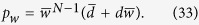


Obviously, 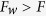 as long as the weak measurement strength 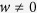. Thus the fidelity of a single copy of noisy W state 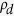 can be indeed enhanced by NRWMs by sacrificing a reduction in the probability. It is easy to see that when 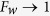 (corresponding to 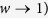, the success probability 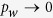.

We now demonstrate that the NRWM can improve the efficiency of distributing the *N*-qubit W state 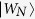 in the AD environment by employing the EDP for amplitude-damped W states. Suppose there are many groups of *N*-qubit amplitude-damped W states 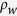. Each group contains two copies, one as the control copy and the other as the target copy. *N* qubits of each copy belong to *N* users (Alice, Bob, Charlie, 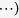, respectively. The W-state distillation protocol is as follows: (i) the *N* users first perform, respectively, a local CNOT gate between two copies of each group, the control copy consists of the *N* control qubits and the target one the *N* target qubits; (ii) they then measure locally the qubits of the target copy in the computational basis 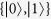; (iii) they keep the control copy if they get the measurement outcome “
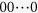
”, and discard it otherwise. Depending on the outcome “
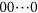
” known through classical communication, the *N* parties share another entangled mixed state


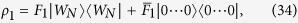


where the fidelity 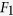 of the noisy W state after the first step of distillation is given by


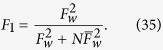


The success probability is


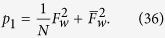


It is easy to prove that 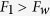 for 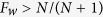. If 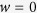, meaning that no NRWM has been performed prior to the distillation operations, only 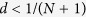 can ensure 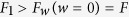. It indicates that the EDP does not work if one directly use the decohered state 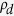, instead of 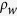 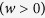, as the input of it when 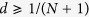. As to the case of 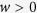, however, the condition of 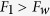 (i.e., 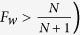 is 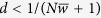. Evidently, the upper bound of *d* in this case could be close to unit by modulating *w* to be near to one. In other words, for any damping rate *d*, the NRWM would enable the above EDP to work, at least in principle, by meeting


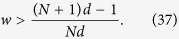


Note that the degree of weak measurement *w* can take any value from 0 to 1, and that the inequality (37) naturally holds for *d*  < 1/(*N* + 1). Moreover, when *d*  ≥ 1/(*N* + 1), the larger *d* is, the larger *w* is required for satisfying the inequality (37).

So, for the case of *d*  ≥ 1/(*N* + 1), the NRWM is evidently beneficial to the distribution of the *N*-qubit W state 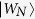, due to the fact that it can decrease the fidelity threshold for distillability of the decoehred W state *ρ*_*d*_ from 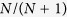 to an arbitrarily small number. Whether the NRWM could still bring benefits in the regime of *d* < 1/(*N* + 1) (keeping it in mind that the EDP can work with the absence of the NRWM under this case)? We next focus on discussing such a problem. It will be shown that the NRWM would contribute to raising the efficiency of the W-state distillation protocol for most values of *d* even in the range 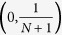, which indicates that the entanglement distribution scheme with NRWM could outperform the scheme without NRWM in most region of *d * 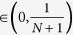.

Based on the success of the first distillation step, the users can carry out the second recurrence step by using 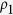 as the input state. By the same token, they can carry on with the third, the fourth, and up to the *m*th recurrence step so that obtaining the nearly perfect W state. In each step, the input states are the states that are kept in the former step with successful events. The fidelity and success probability in each step comply with the recursion formulas (35) and (36) with 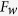 being substituted by the fidelity in the former step. Then after *m* steps, the fidelity 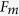 of the obtained state relative to the initial perfect W state 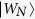 and the corresponding efficiency 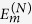 are given by


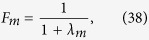



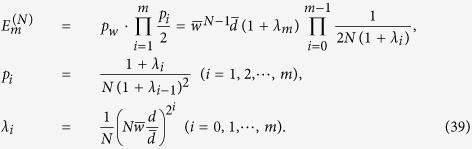


Here 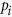 denotes the success probability in the *i*th step. If the fidelity *F*_*m*_  ≥ 1 − *ε*, it means the users obtain a nearly perfect W state denoted by 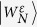 and the W-state distribution succeeds. Following the iteration process as described above, the distribution of the *N*-qubit W state would be accomplished in several steps with finite copies of noisy W state *ρ*_*d*_.

**Figure 5:**
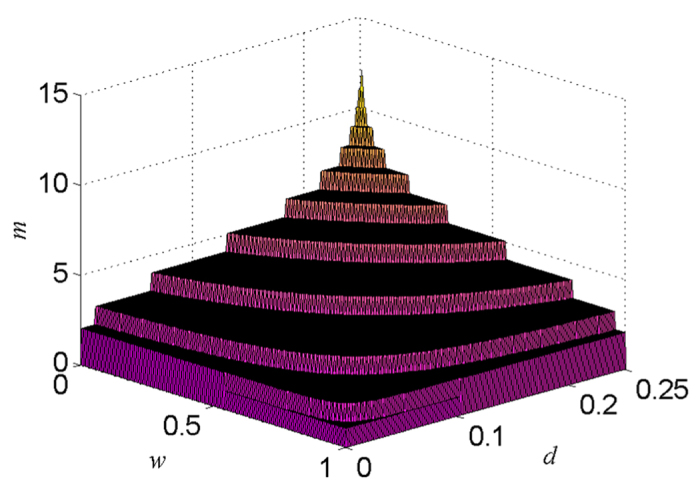
The number of distillation steps *m* needed for finally getting the aim state 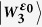.

**Figure 6:**
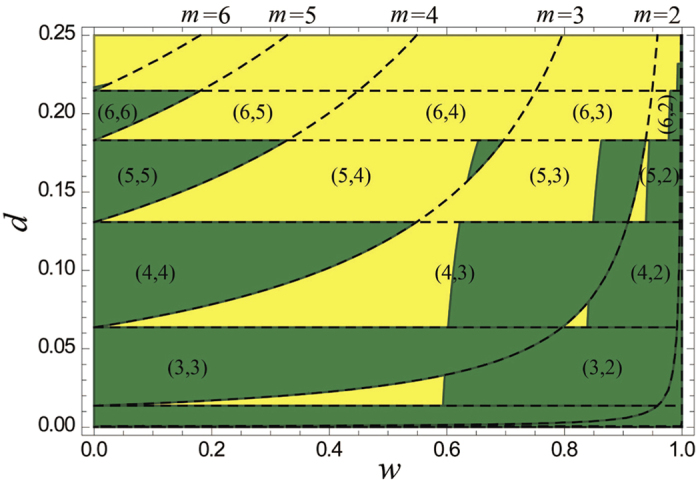
The ranges of *d* and *w* in which *R*^(3)^ >1 (yellow) or ≤1 (green) under the fidelity threshold 1 − *ε*_0_. The dashed curve lines and straight lines correspond, respectively, to 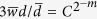 and 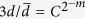 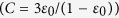 with different *m* (see Methods). The pair-wise numbers 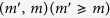 denote that the no-NRWM-scheme and the NRWM-scheme involve, respectively, 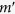 and *m* steps of distillation so that the final fidelity of the noisy W state exceeds the threshold 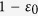 in the encircled regions (see Methods). Note that the no-NRWM-scheme involves only the parameter *d* in the region of 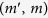.

**Figure 7:**
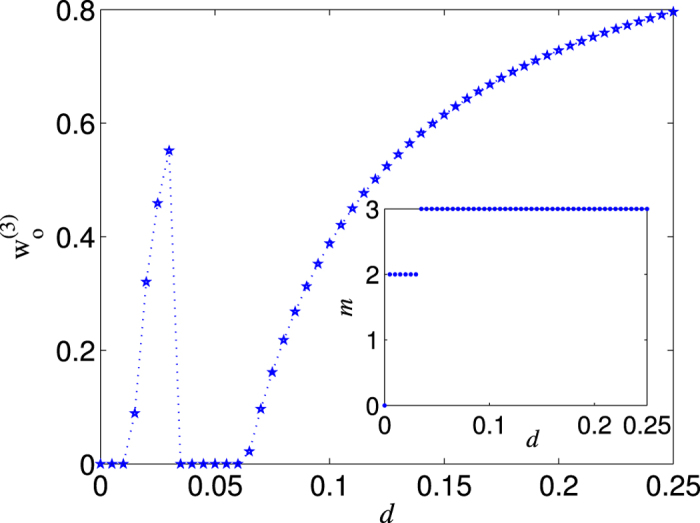
The optimal weak measurement strength 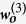 that maximizes the efficiency of getting the nearly perfect W state 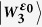. The inset shows the required number of distillation steps *m* for getting 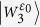 under the optimal degree of weak measurement 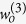.

**Figure 8:**
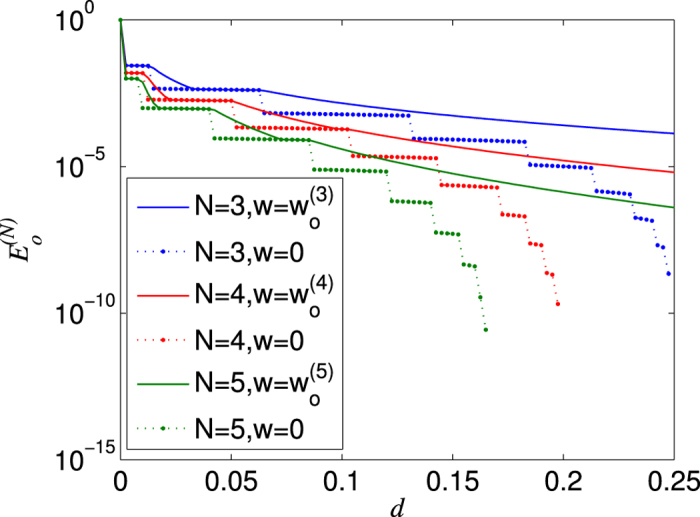
The efficiency 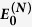 of distributing the nearly perfect *N*-qubit W state 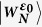 to *N* distant parties in the AD environment. The solid lines denote the optimal NRWM-scheme and the dotted lines stand for the no-NRWM-scheme which works only for *d* < 1/(*N* + 1).

We now take 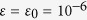 as an example for detailed analysis. For clarity, we first consider 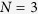. The required number of distillation steps *m* for getting the aim state 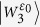 is given in Fig. 5 (see also Methods). From Fig. 5, we can see that for a given *d*, there always exists a region of 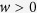 in which the required distillation steps are less than that for the case with 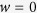. It means that the NRWM can reduce the number of the distillation steps for obtaining the same expected state. The step-wise behavior in Fig. 5 implies that to arrive at the given fidelity threshold, those initial fidelities in a certain region need the same number of iteration steps. This is due to the fact that a smaller initial fidelity may lead to a faster increase in fidelity of the distilled state, which should result from nonlinearity of the iteration formula of fidelity (given in Eq. (35)) and the initial fidelity 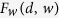 with respect to *d* and *w*. The advantage of the NRWM-scheme in distillation steps can not ensure its efficiency being higher than that of the no-NRWM-scheme. To judge whether the NRWM-scheme could be superior to the no-NRWM-scheme, we need to observe the ratio of the efficiency of the NRWM-scheme 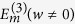 to that of the no-NRWM-scheme 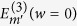, i.e.,


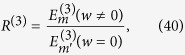


note that 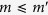 as shown in Fig. 5. The dependence of 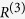 on *d* and *w* is exhibited in Fig. 6, where the region with denotation 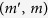 denotes that the no-NRWM-scheme and the NRWM-scheme at least involve, respectively, 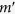 and *m* steps of distillation so that the final fidelity of the noisy W state exceeds the threshold 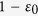. It can been seen from Fig. 6 that the regions of 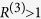 are as follows: (i) (*m*′ > 6, *m* > 2); (ii) (*m*′ = 6, 6 > *m*  ≥ 3); (iii) most part of (*m*′ = 5, 5 > *m* ≥ 3); (iv) about half of 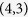; (v) part of (*m*′ ≥ 3, *m* = 2). It implies that when 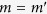 with 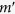 being less than a threshold, *R*^(3)^ ≤ 1. In other cases, however, the regularity of the sign of 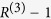 seems to be not clear. It is worth pointing out that the zig-zag behavior in Fig. 6 is well correspondent with the step-wise behavior in Fig. 5. Moreover, the non-ordered phenomenon in Fig. 6 should be related to the fact that 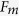 is nonlinear with respect to *d* and *w*, and that 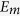 and thus 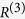 are nonmonotonic with respect to *w*. In a word, the ratio 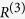 could be larger than one for most values of *d* in the range (0, 1/4). Thus the NRWM-scheme can indeed outperform the no-NRWM-scheme in most region of 0 < *d* < 1/4 in terms of the efficiency. Generally, the larger the degree of decoherence is, the clearer the superiority of the NRWM-scheme. Moreover, the fact that the NRWM is helpful to distributing W states does not mean the larger *w* the better. The optimal weak measurement strength 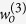 that maximizes the efficiency 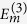 for a given channel damping rate *d* ∈ (0, 1/4) is displayed in Fig. 7, where the inset gives the number of steps *m* needed for getting the aim state 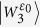 under the case of 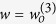. The jump phenomenon in Fig. 7 is matching to 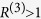 in the bottom yellow region of Fig. 6. With the optimal NRWM, we can compute the best efficiency of 3-qubit W-state distribution 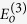 (see Fig. 8).

As for a general *N*, one can still verify that the efficiency of the EDP with NRWM could be higher than that of the scheme without NRWM for most values of *d* in the regime of *d* < 1/(*N* + 1). Furthermore, we can also find the optimal NRWM strength 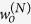 that maximizes the efficiency of extracting a nearly perfect *N*-qubit W state 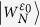 from the decohered state 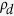 for a given channel damping rate *d*, and then calculate the corresponding highest efficiency 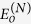. Note that 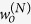 may be dependent on *N*. As examples, we show the optimal efficiencies 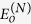 for *N* = 4, 5 in Fig. 8. It can be seen that in the regime of *d* < 1/(*N* + 1), the efficiency of the scheme with NRWM is indeed higher than that of the scheme without NRWM for most values of *d*.

**Figure 9:**
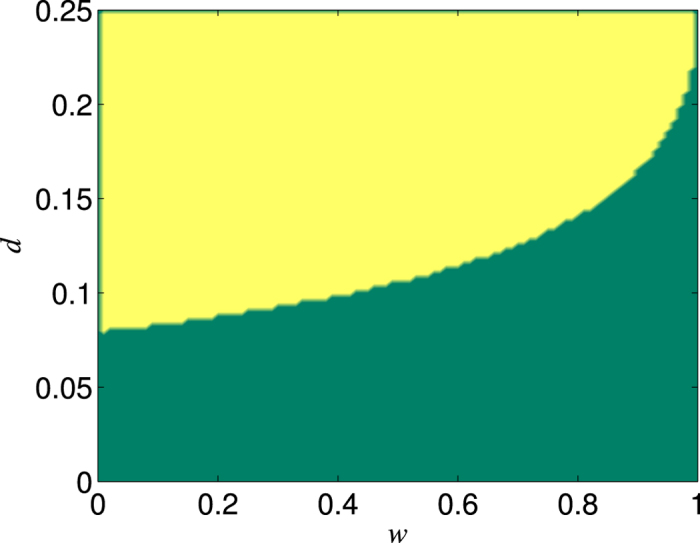
The ranges of *d* and *w* in which 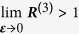 (yellow region) or ≤1 (green region).

Finally, we give a brief discussion on the case of 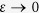. Obviously, 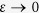 would lead to the fact that the entailed number of distillation steps tends to infinity. Then we obtain (see Methods)


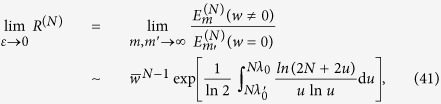


where 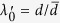. As an example, we give the ranges of *d* and *w* in which 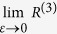 is larger or less than one in Fig. 9. Figure 9 indicates that the ratio 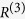 of the efficiency of the NRWM-scheme to that of the no-NRWM-scheme could also be larger than one under the case of 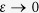 as long as the channel damping rate *d* is not too small. In addition, the larger *d* is, the clearer the advantage of the NRWM-scheme is. The same results could be obtained for *N* > 3.

The positive impact of the NRWM on the W-state distribution could be partly explained by the fact that its positive effect in the distillation phase can surpass its negative effect in the filtering phase when some conditions are satisfied.

## Discussion

Entanglement distillation is a good tool to prepare entangled pure states among distant parties in noisy environments by concentrating the entanglement of a large number of decohered states into a small number of entangled states. Local filtering may be another possible solution to overcoming decoherence of quantum systems. As claimed, a particular filter could be utilized to increase the amount of entanglement of a single-copy noisy entangled state with a ceratin probability. The filtering method, however, cannot be applied for direct production of an entangled pure state. The effect of filtering operations on protecting entanglement from decoherence would be far more exciting if they can be combined with EDPs to improve the efficiency of distributing entangled pure states to distant users who plan to implement remotely faithful quantum information tasks.

In this paper, we have investigated the possibility of improving the efficiency of distilling maximally entangled pure states from entangled mixed states in the AD environment by using the NRWM (a local filtering operation) which has recently been shown to be an effective method for enhancing probabilistically the entanglement of a single-copy amplitude-damped entangled state. We have shown that NRWMs would lead to the decrease of the distillation efficiencies of bipartite maximally entangled states and multipartite GHZ states. However, we found that the NRWM is beneficial to remote distributions of multipartite W states. We demonstrated that the NRWM can not only reduce the fidelity thresholds for distillability of decohered W states, but also raise the distillation efficiencies of W states. The different effects of the NRWM on the distillation efficiencies of W and GHZ states (or bipartite maximally entangled states) may be related to the fact that the former works in an asymptotic way but the latter does not.

Our results indicate that the NRWM is not necessarily helpful to practical entanglement distributions which aim at establishing maximally entangled pure (or nearly pure) states among distant parties, although it can increase to some extent the amount of entanglement of a single-copy entangled mixed state with a certain probability. This leads to a new criterion for measuring the usefulness of a local filter in protecting entanglement from decoherence. These findings are expected to inspire widespread interest on investigating the possibility of improving efficiencies of distributing entangled states in noisy environments by local filtering operations.

## Methods

### Methods of plotting

Explanations on plotting Fig. 6 are given below. The purpose of the distillation is to make the final fidelity of the mixed W state 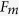 reach to the threshold 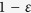 via the minimum number of distillation steps *m*. Thus *m* satisfies 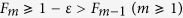. Using Eq. (38), one can readily obtain


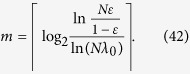


Next, we take 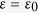 and 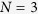 for explaining Fig. 6. With Eq. (42), we can obtain the equations of dashed curve lines in Fig. 6,


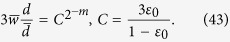


In the region between neighbored two dashed curve lines with *m* and 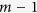, the required number of distillation steps is *m* for the NRWM-scheme. The dashed straight lines parallel to *w* axis can be directly obtained by setting 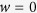 in Eq. (43). Note that 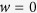 corresponds to the no-NRWM-scheme. So, if 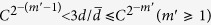, the required number of distillation steps is 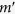 for the no-NRWM-scheme. Then, the region surrounded by neighbored two straight lines and two curve lines satisfies 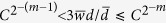 and 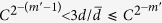, which is denoted by the pair-wise numbers 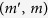 for short. By the way, the intersection points of curve and straight lines satisfy the equation 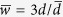. In the region with denotation 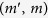 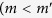, as shown in Fig. 5), the entailed numbers of distillation steps are 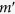 and *m* for the no-NRWM-scheme and the NRWM-scheme, respectively. It should be pointed out that 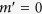 means no purification operation is needed, and that 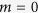 means purification task can be accomplished by only weak measurements. In the regions on and under the curve 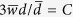, 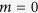. If 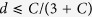, 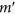 is equal to zero and thus *m* is also equal to zero. For any given 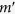 and *m*, the boundary of 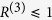 can be obtained by solving, at least in principle, the inequality 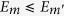, i.e.,


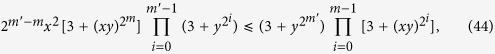


where 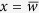 and 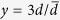. If the inequality has no solution, it means 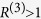 within the total region 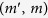.

### Derivation of equation (41)

The derivation of Eq. (41) is given below. When 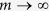, we have


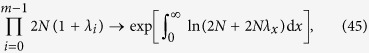



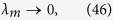


where the inequality *Nλ*_0_ <1 (because 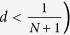 has been utilized. Making two times of variable substitutions 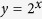 and 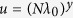, one will get


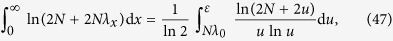


where 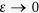. By substituting Eqs. (46) and (47) into Eq. (39), we obtain


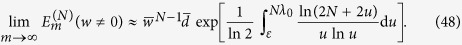


Similarly, we can obtain


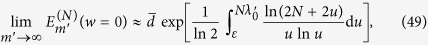


where 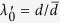. Eq. (41) can be straightforwardly derived from Eqs. (48) and (49).

## Additional Information

**How to cite this article**: Wang X. W. *et al.* Effect of weak measurement on entanglement distribution over noisy channels. *Sci. Rep.* **6**, 22408; doi: 10.1038/srep22408 (2016).
